# The Effects of Spark-Plasma Sintering (SPS) on the Microstructure and Mechanical Properties of BaTiO_3_/3Y-TZP Composites

**DOI:** 10.3390/ma9050320

**Published:** 2016-04-28

**Authors:** Jing Li, Bencang Cui, Huining Wang, Yuanhua Lin, Xuliang Deng, Ming Li, Cewen Nan

**Affiliations:** 1 State Key Laboratory of New Ceramics and Fine Processing, School of Materials Science and Engineering, Tsinghua University, Beijing 100084, China; ljing12@mails.tsinghua.edu.cn (J.L.); cbc14@mails.tsinghua.edu.cn (B.C.); lim@mail.tsinghua.edu.cn (M.L.); cwnan@mail.tsinghua.edu.cn (C.N.); 2 Department of Periodontics, Hospital of Stomatology Wenzhou Medical University, Wenzhou 325027, China; wanghuining1973@gmail.com; 3 Department of Geriatric Dentistry, School & Hospital of Stomatology, Peking University, Beijing 100081, China; kqdengxuliang@bjmu.edu.cn

**Keywords:** spark-plasma sintering (SPS), BaTiO_3_/3Y-TZP, fracture toughness

## Abstract

Composite ceramics BaTiO_3_/3Y-TZP containing 0 mol %, 3 mol %, 5 mol %, 7 mol %, and 10 mol % BaTiO_3_ have been prepared by conventional sintering and spark-plasma sintering (SPS), respectively. Analysis of the XRD patterns and Raman spectra reveal that the phase composition of *t*-ZrO_2_, *m*-ZrO_2_, and BaTiO_3_ has been obtained. Our results indicate that SPS can be effective for the decrease in grain size and porosity compared with conventional sintering, which results in a lower concentration of *m*-ZrO_2_ and residual stress. Therefore, the fracture toughness is enhanced by the BaTiO_3_ phase through the SPS technique, while the behavior was impaired by the piezoelectric second phase through conventional sintering.

## 1. Introduction

As a field-assisted sintering technique, spark-plasma sintering (SPS) has attracted much attention since its advent in the late 1970s [1,2,3]. The starting powders in graphite die are sintered directly instead of being pre-pressed prior to sintering using the conventional processing technique. After graphite die is placed in the furnace, two pistons acting as electrodes load pressure on the upper and bottom surfaces. Due to the good electrical and thermal conductivity of the graphite die, adequate Joule heat is efficiently and quickly transferred to the starting powder under a relatively low voltages. Moreover, the heating rate can be as high as 1000 °C/min, resulting from the adjustable current pulses (milliseconds) [1,2,3].

Both the compressive press and high heating rate work to obtain dense bulks with nano-size grains under a lower sintering temperature, leading to the extensive application of SPS in the dielectric, piezoelectric, and thermoelectric fields, among others [4,5,6,7,8]. Li *et al.* [9] prepared lead-free piezoelectric ceramics Na_0.5_K_0.5_NbO_3_ with 99% relative density at 920 °C by using the SPS technique. It was fairly difficult to obtain when the ceramic sintered in a conventional furnace. Deng *et al.* [10] synthesized nanostructured bulk BaTiO_3_ with a grain size of 20 nm and a relative density of 97% via SPS, while the grain size of BaTiO_3_ was in micrometer range when sintered in a conventional furnace. Furthermore, a series of transparent ceramics [11], such as alumina [12], zirconia [13], and yttrium-aluminum-garnet [14], have been processed with aid of the SPS technique.

Fracture toughness is a highly essential behavior for dental materials [15]. It is believed that the piezoelectric addition could enhance the toughness of ceramics based on the piezoelectric secondary phase toughening mechanism [16,17,18]. Under load, the piezoelectric effect would lead to domain wall motion and dissipate energy in the tips of cracks. Yang *et al.* [19] studied the effect of the piezoelectric second phase, Nd_2_Ti_2_O_7_, on the fracture toughness of Al_2_O_3_, and the toughness increased to 6.7 MPa·m^1/2^. Chen *et al.* [20] prepared a Sr_2_Nb_2_O_7_/3Y-TZP composite and found that the fracture toughness was significant higher than that of 3Y-TZP, as high as 13 MPa·m^1/2^. However, Yang *et al.* [21] found that the addition of BaTiO_3_ suppressed the effect of the transformation toughening of 3Y-TZP, and the fracture toughness decreased instead of increased. Moreover, it has been proven that the electrical charges have a positive effect on the growth and differentiation of osteoblast cells, resulting from preferential adsorption of ions and proteins onto the polarized surfaces [22,23]. As piezoelectric materials could vary surface charges under load, the piezoelectric addition might induce improved bone formation around restorations.

The aim of this study was to investigate effects of the SPS technique on the microstructure, and mechanical properties of BaTiO_3_/3Y-TZP composites as a function of BaTiO_3_ content. The null hypotheses of this study were that SPS would help synthesize dense BaTiO_3_/3Y-TZP bulks with nano-size grains and would improve the mechanical behaviors.

## 2. Results and Discussion

### 2.1. Phase Structure Analysis

**Figure 1:**
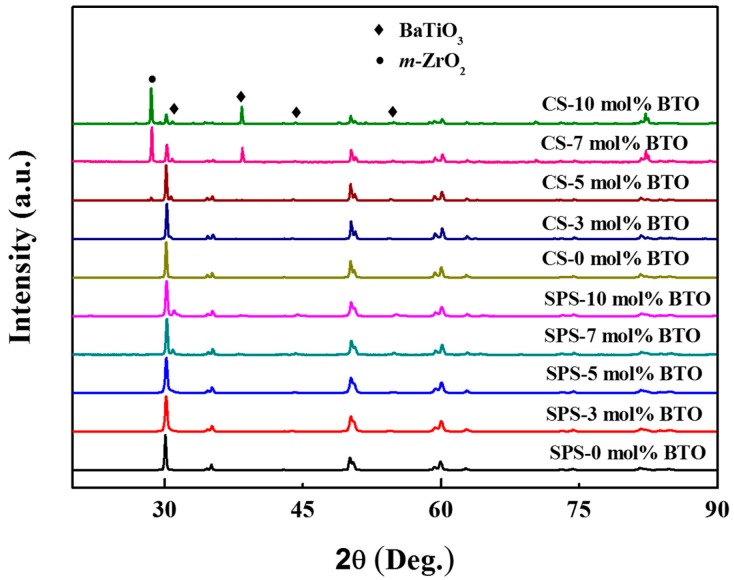
X-ray diffraction (XRD) patterns of spark-plasma-sintered and conventionally sintered BaTiO_3_/3Y-TZP ceramics.

The X-ray diffraction (XRD) patterns of BaTiO_3_/3Y-TZP specimens prepared by different sintering techniques are shown in Figure 1. In this study, CS stands for a conventionally sintered specimen that is sintered in a conventional air furnace. All of the XRD patterns present a crystalline phase of tetragonal ZrO_2_, and the characteristic peaks can be indexed to PDF card #50-1089. With respect to specimens sintered via the SPS technique, diffraction peaks due to BaTiO_3_ were detected as the contents of BaTiO_3_ increased to 7 mol % and 10 mol %. No *m*-ZrO_2_ (monoclinic ZrO_2_) phase was observed. However, as far as conventionally sintered specimens are concerned, peaks can be attributed to BaTiO_3_, and *m*-ZrO_2_ was detected with increasing content of BaTiO_3_. Peaks at 39° can be indexed to the *m*-ZrO_2_ phase and BaTiO_3_ phase. According to the relative intensity of peaks of each phase, it is reasonable to find that the intensity of a peak at 39° mainly originates from the *m*-ZrO_2_ phase. Content of the *m*-ZrO_2_ phase was significantly lower in the spark-plasma-sintered samples; therefore, peaks at 39° seem to be absent in the spark-plasma-sintered samples. Moreover, the dominant ZrO_2_ structure seems to be a monoclinic phase rather than a tetragonal phase when the contents of BaTiO_3_ increase to 7 mol % and 10 mol %, demonstrating a quite different phase structure.

**Figure 2:**
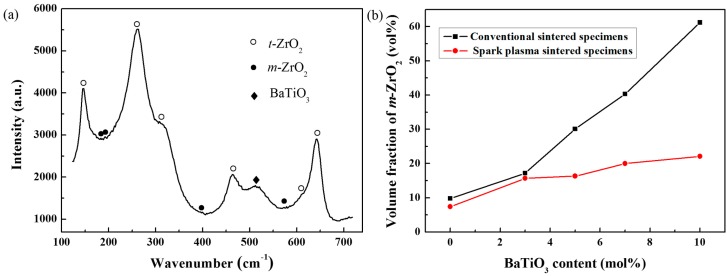
(**a**) Raman spectrum of BaTiO_3_/3Y-TZP (with 7 mol % BaTiO_3_) prepared via the SPS method; (**b**) Volume fraction of *m*-ZrO_2_ content of spark-plasma-sintered and conventionally sintered BaTiO_3_/3Y-TZP ceramics.

Furthermore, the T-M phase transformation within zirconia is investigated by one of the most effective techniques: Raman spectroscopy [24]. Figure 2a shows the Raman spectrum of a representative specimen. Characteristic peaks at wavenumbers 147 cm^−1^, 265 cm^−1^, 464 cm^−1^, and 642 cm^−1^ represent *t*-ZrO_2_, and peaks at wavenumbers 181 cm^−1^ and 190 cm^−1^ reveal the presence of *m*-ZrO_2_ [25]. Figure 2b shows a volume fraction of *m*-ZrO_2_ calculated according to Tabares and Anglada [26]. The content of *m*-ZrO_2_ increases with BaTiO_3_ concentration, which is consistent with the XRD patterns. Compared with conventionally sintered ceramics, spark-plasma-sintered specimens have much lower concentrations of *m*-ZrO_2_, ranging between 4% and 29.4%, whereas the *m*-ZrO_2_ content of conventionally sintered composites varies from 8% to 71.2%. The great discrepancy of the phase structure between specimens may originate from different residual stress states caused by the addition of BaTiO_3_. Less *m*-ZrO_2_ content is better for zirconia ceramics based on the well-known phase transformation toughening mechanism [27]; therefore, the low concentration of *m*-ZrO_2_ might have a positive effect on the fracture toughness of spark-plasma-sintered composites.

### 2.2. Microstructure Analysis

**Figure 3:**
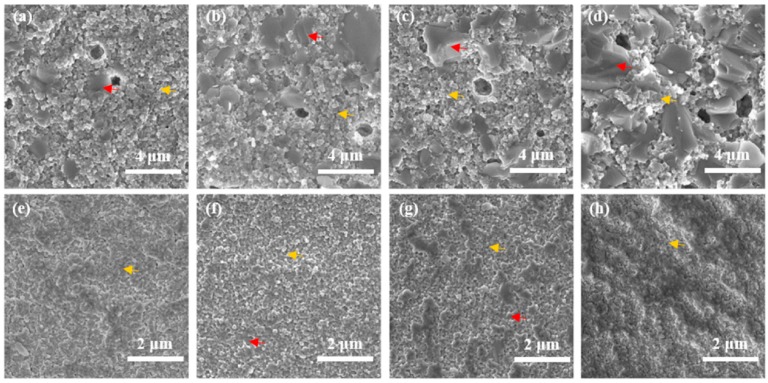
Scanning election microscopy (SEM) images of fracture surfaces of spark-plasma-sintered and conventionally sintered BaTiO_3_/3Y-TZP ceramics. (**a**–**d**) Specimens are prepared by conventional sintering method; (**e**–**h**) Specimens are prepared via the SPS method. (**a**,**e**) 3 mol % BaTiO_3_; (**b**,**f**) 5 mol % BaTiO_3_; (**c**,**g**) 7 mol % BaTiO_3_; (**d**,**h**) 10 mol % BaTiO_3_. The red arrow denotes the BaTiO_3_ phase and the yellow arrow denotes the 3Y-TZP phase.

Figure 3 shows scanning election microscopy (SEM) images of spark-plasma-sintered and conventionally sintered BaTiO_3_/3Y-TZP ceramics as a function of BaTiO_3_ content. The As for conventionally sintered composites, two different kinds of grains, ZrO_2_ and BaTiO_3_ are clearly observed. With increasing amounts of BaTiO_3_, the grain size of ZrO_2_ is about 200 nm, remaining substantially unchanged, while grain size of BaTiO_3_ increases from 1 to 3.5 μm with the BaTiO_3_ content. Since grain size of BaTiO_3_ is 5 to 17.5 times larger than that of ZrO_2_, the mismatch of grain size leads to pores in ceramics and might introduce stress between grains, resulting in the stress-induced T-M phase transformation. Hence, the content of *m*-ZrO_2_ increases, as shown in Figure 2. Compared with conventionally sintered composites, spark-plasma-sintered ceramics exhibit significantly smaller grain sizes, especially for BaTiO_3_ grains, which can be attributed to the compressive press and the high heating rate. Moreover, with close ion radii and the same valence, it is likely for Ti^4+^ to partially substitute Zr^4+^, forming a solid solution, (Zr,Ti)O_2_ [21]. Thus, spark-plasma-sintered ceramics show traces of liquid-phase sintering with increasing content of BaTiO_3_, resulting in more dense composite bulks [28]. The larger grain size for higher BaTiO_3_ content in conventionally sintered specimens may also support this conclusion.

According to the relative density, bulk porosities of composites (Table 1) are calculated by using the following equation: *P* = (1 − *ρ*) × 100%, where *P* is the bulk porosity, and *ρ* is the relative density. With increasing amounts of BaTiO_3_, porosity trends between conventionally sintered specimens, and spark-plasma-sintered composites vary greatly. Porosity increases with BaTiO_3_ content, due to the mismatch of grain size in conventionally sintered ceramics. By contrast, both of the slight mismatches in grain size and the liquid-phase sintering produce effects on the spark-plasma-sintered composites; therefore, porosity values float slightly. However, it is reasonable to find that spark-plasma-sintered composites are denser than the conventionally sintered ones, which are in consistency with the observed SEM images.

### 2.3. Mechanical Properties

**Figure 4:**
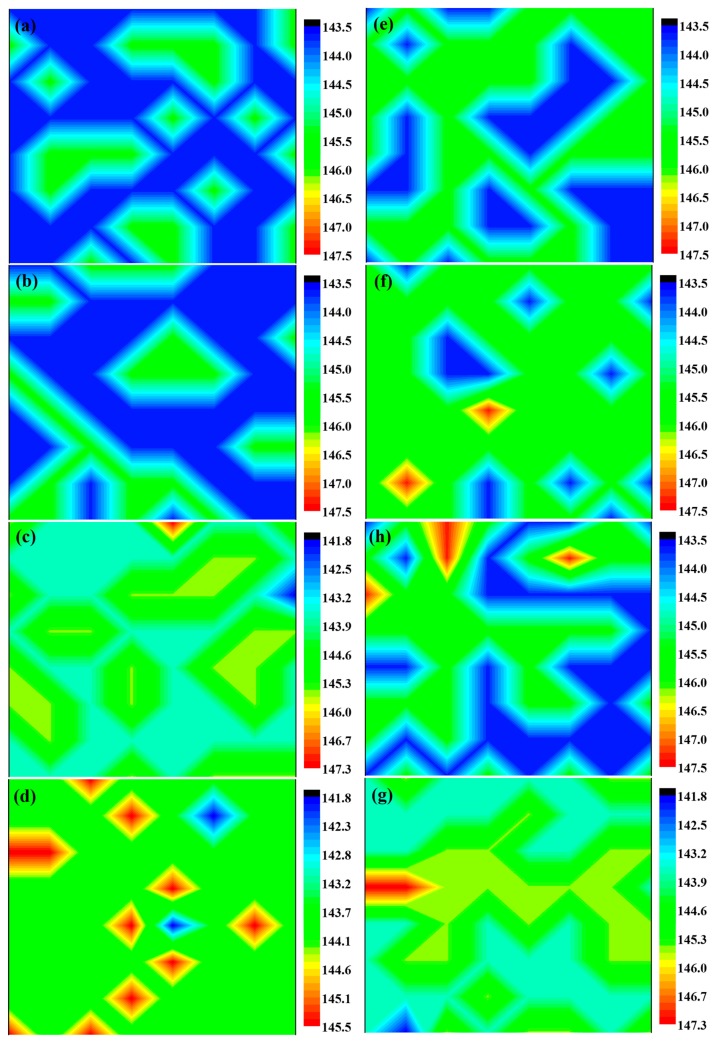
Quantitative Raman maps of spark-plasma-sintered and conventionally sintered BaTiO_3_/3Y-TZP ceramics. (**a**–**d**) Specimens are prepared by conventional sintering method; (**e**–**h**) Specimens are prepared via the SPS method. (**a**,**e**) 3 mol % BaTiO_3_; (**b**,**f**) 5 mol % BaTiO_3_; (**c**,**g**) 7 mol % BaTiO_3_; (**d**,**h**) 10 mol % BaTiO_3_.

The residual stress state of the composites could be effective for not only the phase transformation but also the fracture toughness. As an attempt to depict the residual stress state, Raman maps of each specimen (5 × 5 μm^2^) were recorded, and the data were analyzed by MATLAB software. Specimens without BaTiO_3_ serve as the control group, and the mean wavenumber is 145.5 cm^−1^. A shift of the peak toward higher wavelength number indicates the presence of residual compressive stress, which helps crack closure. By contrast, a peak shift toward lower wavelength number reveals the presence of residual tensile stress. Moreover, a larger peak shift means a higher residual stress. As seen in Figure 4, a mixture of tensile and compressive stress is recorded in spark-plasma-sintered composites, but significantly more tensile stress is found in the conventionally sintered specimens. Moreover, the peak shift is larger for conventionally sintered specimens with more BaTiO_3_ content.

**Figure 5:**
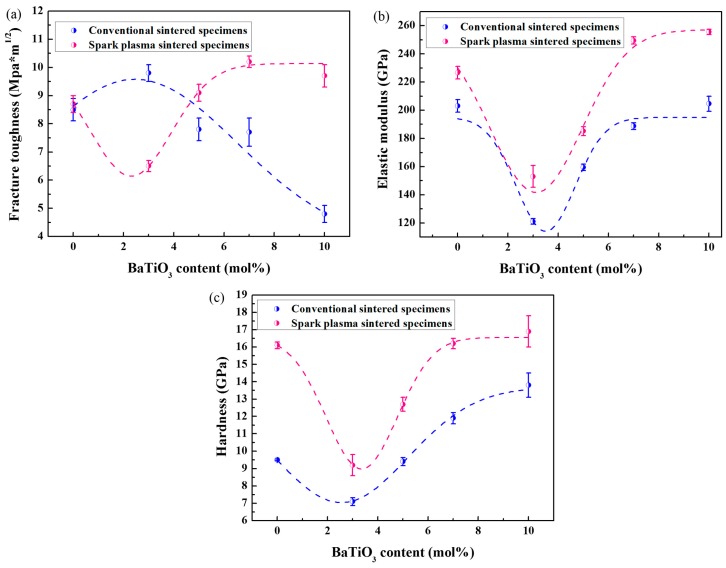
The mechanical properties of specimens with different BaTiO_3_ contents. (**a**) Fracture toughness; (**b**) elastic modulus; (**c**) hardness.

Figure 5 shows the fracture toughness, Vickers hardness, and elastic modulus values as a function of BaTiO_3_ content. Even though the addition of BaTiO_3_ would enhance the fracture toughness through the piezoelectric effect, both the accompanied high porosity and the *m*-ZrO_2_ content have negative effects on the behavior of specimens prepared by conventional sintering. Therefore, the fracture toughness greatly trends downward after increasing slightly. Owing to the compressive press and the high heating rate of the SPS technique, the grain size of BaTiO_3_ is quite smaller, and ceramics show traces of liquid-phase sintering, resulting in more dense composites and a low concentration of *m*-ZrO_2_. Thus, the expected coupling effects of the piezoelectric secondary phase toughening mechanism and phase transformation toughening mechanism lead to a high toughness of the specimens prepared via the SPS method. As for composites with 3 mol % BaTiO_3_, the fracture toughness of the conventionally sintered specimen is significantly higher than that of the spark-plasma-sintered specimen, which may be attributed to the difference in the effect of the piezoelectric secondary phase toughening mechanism, resulting from different contents of the BaTiO_3_ phase. XRD patterns of both specimens show no BaTiO_3_ phase, but SEM images reveal the existence of BaTiO_3_ grains in the conventionally sintered specimen (Figure 3a). The addition of 3 mol % BaTiO_3_ in the spark-plasma-sintered specimen may serve as a doping agent rather than a polycrystalline phase, which destroys the fracture toughness.

Both the elastic modulus and hardness show a similar trend with increasing amounts of BaTiO_3_. The as-prepared BaTiO_3_ has a lower elastic modulus (~72.8 GPa) and hardness (~1.4 GPa) than does 3Y-TZP, but there is no expected decline in the composites. After decreasing at first, these two behaviors increase with BaTiO_3_ content, which may be attributed to the formation of the solid solution, (Zr,Ti)O_2_. As spark-plasma-sintered ceramics suffer a high compressive press during sintering, they show more traces of liquid-phase sintering, suggesting more amounts of solid solution [29]. Therefore, these specimens reveal a higher elastic modulus and hardness, compared with the conventionally sintered specimens.

## 3. Materials and Methods

### 3.1. Materials

3Y-TZP (TZ-3YSB-E, Tosoh Co., Tokyo, Japan) with an average particle size of 90 nm and BaTiO_3_ (Sinopharm Chemical Reagent Co., Shanghai, China) with an average particle size of 100 nm were used to prepare the BaTiO_3_/3Y-TZP composite.

### 3.2. Preparation of Porous Zirconia Ceramic

The starting materials, 3Y-TZP and BaTiO_3_, at 0 mol %, 3 mol %, 5 mol %, 7 mol %, and 10 mol % were mixed together by alcohol-based ball milling for 12 h, respectively. The mixture powders were dried in oven for sintering. Some of the mixtures were pressed at a pressure of 4 MPa, followed by a cold isostatic pressing at 200 MPa, and the samples were then heated up to 1400 °C at a rate of 100 °C/h and kept for 2 h in a conventional air furnace. Some of the mixtures were sintered directly via SPS. The heating rate was 110 °C/s, and the sintering temperature was 1175 °C.

### 3.3. Characterization

X-ray diffraction spectroscopy (Rigaku, D/MAX-2550V, Tokyo, Japan) was employed to analyze the phase composition. Morphologies of fracture surfaces were examined via SEM (Hitachi, S-2500N, Tokyo, Japan). The relative density was measured by the Archimedes method. The volume fraction of monoclinic ZrO_2_ was measured by a Raman spectrometer ((Hiroba, LabRAM HR Evolution, Tokyo, Japan). It was calculated based on the equation:

(1) $$V_{m}=\frac{I_{{}_{m}}^{181}+I_{{}_{m}}^{190}}{0.32\left(I_{t}^{147}+I_{t}^{265}\right)+I_{{}_{m}}^{181}+I_{{}_{m}}^{190}}$$

where *I_t_* and *I_m_* are the integrated intensities of the tetragonal and monoclinic peaks, respectively. A nano-indentation tester (MTS, Palo Alto, CA, USA) was applied to analysis of the Vickers hardness and elastic modulus. The hardness was calculated by the equation:

(2) $$H_{V}=1.8544P/d^{2}$$

where *H_V_* is the Vickers hardness; *P* is the load; and *d* is the diagonal of the indentation. The elastic modulus was further inferred by using the equation:

(3) $$E=0.45H_{V}/\left(a/b-a/b_{1}\right)$$

where *E* is the elastic modulus; *b* is the length of the shorter diagonal; *b*_1_ is the length of the longer diagonal; and *a* is the length of the crack. A universal test instrument (Shimadzu, EZ-100, Tokyo, Japan) was employed to measure the fracture toughness of specimens by the single-edge-notched beam method with a loading rate of 0.05 mm/min. Bending bars (*n* = 12) per specimen were cut into 2 × 4 × 16 mm^3^ with a diamond blade, and the notch depth was approximately 2 mm. The fracture toughness was calculated using formula:

(4) $$K_{IC}=\frac{P_{0}\times l}{BW^{3/2}}f(\frac{a}{W})$$

where *P*_0_ is the load; *l* is the span; *B* is the height of bar; *W* is the width of the bar; and *a* is the depth of the notch.

## 4. Conclusions

A series of BaTiO_3_/3Y-TZP ceramics have been prepared by conventional sintering and SPS, respectively. The phase structure, microstructure, and mechanical properties of the composites were investigated as a function of BaTiO_3_ content. Our results show that the SPS technique has a remarkably positive effect on the behaviors of BaTiO_3_/3Y-TZP composites. Spark-plasma-sintered specimens are superior in fracture toughness due to the coupling effects of the piezoelectric secondary phase toughening mechanism and the phase transformation toughening mechanism. These results reveal that the piezoelectric secondary phase, BaTiO_3_, could enhance the fracture toughness of zirconia through the SPS technique.

## Figures and Tables

**Table 1:** The porosity of spark-plasma-sintered and conventionally sintered BaTiO_3_/3Y-TZP ceramics.

BaTiO_3_ Content	0 mol %	3 mol %	5 mol %	7 mol %	10 mol %
Porosity (%) (CS)	2.7	5.9	9.6	12.5	14.8
Porosity (%) (SPS)	0.5	4.8	3.3	1.5	3.2
